# Decision Making in Plants: A Rooted Perspective

**DOI:** 10.3390/plants12091799

**Published:** 2023-04-27

**Authors:** Jonny Lee, Miguel Segundo-Ortin, Paco Calvo

**Affiliations:** 1 Minimal Intelligence Laboratory (MINT Lab), University of Murcia, 30100 Murcia, Spain; leejonathan.cw@gmail.com (J.L.); fjcalvo@um.es (P.C.); 2 Department of Philosophy, University of Murcia, 30100 Murcia, Spain

**Keywords:** decision making, plant behaviour, bacteria, intelligence

## Abstract

This article discusses the possibility of plant decision making. We contend that recent work on bacteria provides a pertinent perspective for thinking about whether plants make choices. Specifically, the analogy between certain patterns of plant behaviour and apparent decision making in bacteria provides principled grounds for attributing decision making to the former. Though decision making is our focus, the discussion has implications for the wider issue of whether and why plants (and non-neural organisms more generally) are appropriate targets for cognitive abilities. Moreover, decision making is especially relevant to the issue of plant intelligence as it is commonly taken to be characteristic of cognition.

## 1. Introduction

At the centre of debates over plant intelligence lies the question of whether plants possess cognitive abilities, such as learning, memory, numerosity, anticipation, and so on [1,2,3,4]. This paper focuses on plant decision making [5] and connects it with the widespread discussion of decision making in non-neural organisms. Generally speaking, an organism is said to make a decision whenever (i) it selects between alternative courses of action, and (ii) this selection is not random but is based on an evaluation of the alternatives in light of some collected information [6]. We contend that recent work on bacteria provides a pertinent perspective for thinking about whether plants make choices. Specifically, the analogy between certain patterns of plant behaviour and apparent decision making in bacteria provides principled grounds for attributing decision making to the former. Though decision making is our focus, the discussion has implications for the wider issue of whether and why plants (and non-neural organisms more generally) are appropriate targets for cognitive science. Moreover, whilst we avoid defending any position on the wider implications for plant intelligence, we note that decision making is commonly taken to be characteristic of cognition (e.g., [7], but see [8]) and is therefore pertinent to debates about plant intelligence.

We begin by introducing the notion of decision making and outlining recent work on bacteria (Section 2). We then turn to prima facie evidence for decision making in plants before discussing one reason to think that the analogy between single-celled organisms and plants does not hold, namely, because plants do not genuinely select between behaviours (Section 3). We close by forecasting the importance of future research (Section 4).

## 2. Decision Making in Bacteria (and Beyond)

As already mentioned above, decision making involves selecting between several possible options for behaviour based on information about the organism and/or its environment (e.g., see [9,10,11]). A perennial problem with assessing whether some atypical taxa (such as plants) exhibit a cognitive phenomenon (such as decision making) is defining the ability in question. Nevertheless, we take this generic characterisation to be sufficiently ecumenical as a starting point. The more liberally minded may insist that decision making need not be ‘behavioural’ but also expressible via physiological or cognitive changes (e.g., [6]). Although we do not preclude a broad definition of behaviour that encompasses physiological/cognitive changes, we must note that the notion of ‘behaviour’ is itself highly contested (see Section 3.2).

Before examining whether plants undertake decision making, it will be fruitful to turn first to established research on bacteria, insofar as this will furnish us with a clear-cut phylogenetic entry point as we transition from bacterial unicellularity into the acquisition of plant multicellularity, and from prokaryotic into eukaryotic forms of life. The first unicellular eukaryote is thought to have resulted from bacterial genome fusion and synergistic interactions between, probably, cyanobacteria and proteobacteria ancestors [12]. Subsequently, according to phylogenetic reconstruction, two bacterial endosymbiotic events resulted in the origins of the precursors of mitochondria and chloroplasts [13]. First, the uptake of an alpha-purple bacterium marked the origin of the mitochondria in the common ancestor of plants and animals, and at a later stage, the uptake of a photosynthetic cyanobacterium paved the way for chloroplasts, this time, exclusively in the plant lineage. Plants, therefore, presented an evolutionary innovation, whereas the rest of the eukaryotic life forms (up to and including humans) preserved their ancestral cellular organization [14]. One way or another, it is highly unlikely that a previously evolved adaptive trait is jettisoned at a later stage [15].

Following the principle of evolutionary conservatism, it is worth noting that the evolutionary origins of eukaryote neurobiology run very deep in the tree of life with many neural-based aspects of cognition already present in bacteria, serving to channel their cellular processes of survival (e.g., neural network-like signal transduction in bacteria) [16]. In a similar vein, the number of structural and functional similarities between neurons and plant cells being researched keeps growing [17]. Several proteins known to mediate neurotransmission synaptically in animals have been found in bacteria, throwing light upon the phylogenetic development of neurotransmitters; glutamate and gamma-aminobutyric acid (GABA) are among the chemicals that function, not as mere metabolites, but rather as plant signalling molecules (‘biomediators’, in plant physiological parlance to distinguish them from animal neurotransmitters). In addition, actin and other cellular motors are also found in plants [18].

It is increasingly common to claim that bacteria are capable of elementary forms of decision making. Among the supporting evidence is the discovery of ‘control mechanisms’ underlying locomotion. These are distributed, ‘heterarchichally structured’ mechanisms for obtaining information about the organism’s internal and external conditions that facilitate the evaluation of alternative behaviours and the selection between them [19]. The efficacy of control mechanisms for producing adaptive behaviour is exemplified by locomotive chemotaxis in *E. coli*. In brief, these bacteria are faced with selecting between directions for locomotion, relying on their flagella (the hair-like structure protruding from the cell body) attached to a motor for moving around, and travelling up or down gradients of different substances that attract or repel them. The motor rotates either clockwise—which moves the organism forward—or counterclockwise—which causes the organism to tumble and turn to face another direction. These behaviours are not triggered randomly or as a simple reaction to perturbation. Rather, they are the result of ‘control mechanisms’ that gather information and, equally important, evaluate that information to govern ‘production mechanisms’ (those responsible for the behavioural output) [4]. In particular, *E. coli*, as well as many other bacteria, use a two-component regulatory system (TCS) [20], functionally similar to the nervous system of animals, which serves the role of a memory and inner connection between sensors and effectors. Courtesy of this system, *E. coli* can take sequential measurements of the substance concentration whose net result is a systematic form of chemotaxis [21]. These control mechanisms, however minimal, are adequate for adaptively determining between different possible actions. It is for this reason that many theorists attribute a form of decision making to bacteria.

As *E. coli* demonstrate, the primary appeal of attributing decision making to bacteria is their ability to switch between behaviours based on the receipt and evaluation of information, which resembles decision making in more paradigmatic cases (corresponding to our initial characterisation, above). Furthermore, describing such behaviour selection in bacteria as a form of decision making suggests a generic, non-idiosyncratic (non-taxa specific) notion. This is attractive because it implies that more-or-less similar abilities (i.e., ‘decision-making abilities’) may be identified and compared across different branches on the tree of life (see Section 3.2 below for related discussion).

As Bechtel and Bich explicate, decision making is ‘an activity that all organisms as autonomous systems must perform to keep themselves viable […] [g]iven the variable nature of the environment and the continual degradation of the organism’ [22] (p. 1). In keeping with the bacteria case, the production of flexible behaviour required to survive in a dynamic environment requires organisms to regulate processes of production using mechanisms of control that measure environmental variables and evaluate the resulting information regarding certain standards (or ‘norms’) of viability. However, control mechanisms are not always hierarchal (i.e., mechanisms organised into successively higher-level control mechanisms) but typically heterarchical. In effect, control mechanisms can function with (more-or-less) independence in the absence of a centralised controller. In short, the case of bacteria demonstrates how selecting between different possible behaviours based on the receipt and evaluation of information according to certain norms of viability is possible without a centralised ‘executive’ mechanism. Notice that whilst an approach such as that advocated by Bechtel and Bich permits decision making to be widespread—allowing even single-celled organisms to make choices—it does not trivialize the concept, e.g., allowing every biological process to count as decision making. Rather, decision making involves identifiable (if highly distributed) mechanisms of control that measure and evaluate environmental variables.

A first-pass objection to the idea of decision making in bacteria is the assumption that the ability depends on the authority of an executive mechanism. Such a view likely results from modelling decision making on deliberative, conscious choices in humans, where familiar decisions at least seem to be determined by a centralised controller.

However, it is debatable whether the assumption holds in most forms of decision making. For instance, the medicinal leech (*Hirudo verbena*) selects between swimming and crawling but does not depend on a centralised neural mechanism, but rather on the emergent effect of 21 independent ganglia located between its ‘head and tail brains’ [23] (p. 3). Similarly, extensive work on domesticated cats, for example, has demonstrated that decision-making mechanisms in neural organisms with brains are distributed across cortical and subcortical structures. The neural circuitry responsible for decision making in these cases is critically modulated by a range of often broadly diffused chemical signals carrying information about the state of the environment and organism [19] (p. 1061). Brains, so the evidence shows, do not obviate heterarchical organisation, at the very least. In fact, some human behaviour may emerge from the coordinated activity of heterarchical control mechanisms as well (for extended discussion, see [22,23]).

In summary, even neural organisms rely on decentralised mechanisms and non-neural components when making decisions. One could, of course, still insist that only deliberative decision making of the sort familiar to human introspection is bona fide decision making, hence any similarities between processes in bacteria (or leeches) and human decision making remain superficial when it comes to determining cognitive abilities. We note that this position leads to an excessively restrictive notion of decision making that would exclude even paradigmatic cases of non-conscious decision making in humans which are standardly accepted by cognitive science (e.g., see [10]; see Section 4 below for related discussion).

A related worry stemming from a ‘cognitivist’ approach is that any genuine cognitive ability must be underwritten by a representational process [24,25]. Hence, for non-neural organisms to make genuine choices in the same (cognitive) sense, it is necessary for them to trade in representations. One might argue that this is the case [26]. However, it is worth noting that cognitivism is no longer the default assumption in the field, and many would reject its conception of cognition nowadays. We cannot delve into these murky issues here. However, notice that even if the elementary forms of decision making surveyed in this paper are not considered bona fide cognition, then the ramifications for understanding the role and distribution of decision-making abilities in the tree of life remain ambiguous; if not all ‘decision making’ is *truly* cognitive, perhaps *true* cognition is less vital than first thought.

## 3. Making Our Minds up about Plant Decision Making

Like bacteria and all other organisms, plants face myriad challenges to survival in an unpredictable world. To meet these challenges, plants must continually adapt to their dynamic surroundings by growing flexibly, deploying a range of defence mechanisms, and managing the uptake and distribution of nutrients. Given that plant physiology, like all physiology, incurs energetic costs, plants must constantly prioritise where to grow, which defence mechanisms to trigger, and what resources to favour. On the face of it, it is reasonable to conclude that plants must make choices too. In Section 3.1 we dig deeper into the idea of plant decision making. In Section 3.2 we discuss reasons one might remain sceptical.

### 3.1. A Potted Introduction to Plant Decision Making

Evidence for plant decision making can be found above and below ground [27]. Well-known above-ground examples are the dodder plant (*Cuscuta pentagona*) [28] and the tropical vine *Monstera gigantea* [29]. Given the choice to parasite a tomato plant (*Lycopersicon esculentum*) or a wheat seedling, the dodder plant will grow toward the former, rejecting the lower quality and less appealing wheat host. However, if wheat is the one and only option available in the vicinity, dodder will grow towards it, although more slowly and growing fewer tendrils [15]. In the case of *Monstera*, young seedlings can tell light and dark patches apart, growing toward the former, as dark patches correspond to the base of the trunks of potential hosts [29]. As the host is reached, *Monstera* seedlings will switch their skototropic, dark-oriented behaviour for a phototropic pattern of upward climbing.

Because these examples have been discussed at length, our focus in this section will be on the less well-known root growth (for similar discussions of decision making at the shoot level see [28,30,31,32,33,34]. Take, for instance, the so-called ‘binary decision making’ of maize roots [35]. When maize (*Zea mays* L.) roots reach the fork of a Y-maze (a growth space with the shape of an inverted Y), they can grow down one arm or the other. Unsurprisingly, in the absence of volatiles roots exhibit no preference, using only gravitational direction to determine growth. However, when a gradient of volatiles is introduced, roots are repelled or attracted, as inferred from their differential patterns of growth towards or against particular chemical gradients. If exposed to, say, diethyl ether or ethylene in one arm, roots will grow towards it; by contrast, exposition to methyl jasmonate in one arm will trigger an escape tropism, similar to the type of photophobic, avoidance behaviour [36] or halotropic (salt-stress) responses [37] observed in roots. More striking, root growth appears dependent on the combination of environmental conditions such as chemical volatiles, indicating ‘that the different combinations of types/concentrations of diverse volatiles affect the root decision making’ [35].

The sensitivity of root growth to combinations of environmental conditions instead of single factors has also been found, for example, in the preference of *Calamgrostis canadensis* for light plus warm soil over other combinations [5]. Forced choices between hydrotropism and root gravitropism for differing moisture gradients under the gravity pull have also been reported [38]. Note, in addition, that increased growth in one part of a plant’s root network is frequently accompanied by decreased growth in another, indicating that plants coordinate root growth across the whole organism [39,40]. This implies that plants engage in a sort of trade-off evaluation, where the growth of some structures is prioritized over others in relation to the current needs.

Consider this other example. When grown alone, the roots of *Abutilon theophrasti* will distribute broadly and uniformly regardless of whether the nutrient distribution is heterogenous or homogenous [41]. When a competitor is introduced and nutrient distribution is homogenous, roots grow more selectively, avoiding contact (and thus, competition) with neighbouring roots. However, when another exemplar is introduced and nutrient distribution is heterogenous, roots exhibit reduced selectivity, and an increased tendency to grow in areas shared with neighbouring roots. This shows that growth patterns are dependent on integrating information about nutrients and neighbours. More generally, root growth patterns seem to rely on the detection and integration of myriad signals carrying resource and non-resource information [42]. Further work indicates that some plants discriminately distribute more resources to parts of roots in patches of soil with increasing levels of nutrients over those in areas with higher absolute but non-increasing levels of nutrients, meaning the plant root growth is sensitive to temporal change as well [43,44].

Finally, pea plants switch between risk-prone or risk-averse root growth depending on context. Dener et al. [45] grew split-root pea plants in such a way that their root tips could grow into separate pots in two conditions, sharing equal mean nutrient irrigation; in one condition, the pots contained constant levels whilst the other contained fluctuating concentrations. The study supported the conclusion that pea plants preferred soil with variable distribution in the context where mean nutrient levels were sufficiently low but constant distribution where mean nutrient levels were enough to meet their metabolic needs. The authors took this to demonstrate risk sensitivity, switching between risk-prone and risk-averse growth as a function of resource availability, congruent with predictions from risk sensitivity theory (for further discussion on the ‘rationality’ of root growth patterns, see [46]).

This small sample of the empirical literature suggests that when confronted with a dynamic and heterogeneous environment, plants adaptively select between growth patterns based on information about their environment. In other words, plants seem to choose where to grow in a way that suggests a sort of normative evaluation.

Compared with bacteria, the mechanisms for such apparent decision making in plants are less certain (in part because their physiology is more complex, with processes spanning across the cellular level—say, touch receptors—and the levels of both organs and organism—say, sensitive cells and sensitive hairs, respectively [27]) and harder to generalise (because their physiology varies more across species). However, a sketch is possible: plants achieve behaviours such as selective root growth in response to the environment by exploiting receptors sensitive to a range of stimuli (akin to animals), distributed internal electrical and chemical signalling systems for information integration (akin to single-celled organisms and animals in some cases), and mechanisms for organism-level behaviour, often through phenotypic changes via gene expression (e.g., [47]). This contrasts with the view that plant behaviour is purely genetically determined by natural selection or epigenetically determined by the environment (e.g., [48]).

In summary, though many details are still lacking, plants appear capable of organism-level decision making through distributed mechanisms, such as bacteria. We say ‘appear’ because one may harbour lingering doubts as to whether the analogy between plants and bacteria holds because only bacteria select between genuine behaviours. We deal with this objection in the following section.

### 3.2. Growing Pains

With the aid of a microscope, one can appreciate the buzz of bacterial activity. However, gazing at a potted cactus or strip of grass, plants can appear tediously immobile. Compared with bacteria, it is harder to think of plants as behaving, and one might insist that, unlike the former, plants do not selectively move by integrating information. In this section, we offer an answer to both concerns. First, we argue that it is not clear that movement is required for behaviour. Second, we contend that plants, like bacteria, do select between movements, albeit (a) at a slower time scale and (b) primarily via phenotypic plasticity (e.g., patterns of growth), rather than locomotion. Taking into account the evidence surveyed above, we hold that the analogy between bacteria and plants is strengthened: both select between movement-based behaviours (*mutatis mutandis*) based on the evaluation and integration of information via distributed (non-centralised) mechanisms. Thus, if one grants decision making to bacteria, one ought to grant decision making to plants.

‘Behaviour’ is a notoriously vague concept, with disparate definitions found across disciplines. In responding to this ambiguity, Levitis et al. [49] propose a discipline-neutral definition based on a meta-study of responses across biology: ‘behaviour is the internally coordinated responses (actions or inactions) of whole living organisms (individuals or groups) to internal and/or external stimuli, excluding responses more easily understood as developmental changes’ (p. 103). This non-idiosyncratic definition comfortably encompasses plants alongside bacteria. Notice, however, that the definition does not depend on movement; if plants do not move, they are not thereby excluded from behaving. Rather, what matters is whether organisms internally coordinate actions and our examples above suggest that plants do. Thus, taking such a characterisation for granted, there is no reason to deny decision making to plants on the basis that they do not move [50].

However, even if one insists on a more restrictive definition of behaviour that required movement (e.g., [51]), we see no reason to exclude plants [5,52] either. The idea that plants move, via idiosyncratic means, stretches at least as far back as Darwin (for example, see ‘The Power of Movement in Plants’; [53]). Darwin appreciated that plants are constantly in motion (for a book-length tribute to the pioneering work of Darwin, see [15]). Of course, plants do not locomote. Instead, plants primarily achieve motion via directional growth responses to the environment (such as phototropism and gravitropism), as well as non-directional movements that are typically regulated by turgor pressure or electrical stimulation (such as thigmonasty and thermonasty). Some plant movement is incredibly fast; *Mimosa pudica* folds its leaves in response to touch in around 5 s, whilst Venus flytraps (*Dionaea muscipula*) close their traps around 100 ms (neither are growth-based movements). However, most plant movement is growth-based and slow compared with animal movement, and imperceptible to the human eye. This likely goes some way to account for our tendency to think of plants as stationary. The stark reality of plant motion is laid bear with timelapse photography which allows plant motion to be perceptible at our timescale. Timelapse photography does for our appreciation of plants what microscopes do for our appreciation of bacteria.

Plants thus move slowly and largely by growth but, following Darwin, they do move. Thus, even if decision making requires selecting between movements, then plants are not excluded from decision making. The analogy between bacteria and plants is saved. To be clear, the claim is not that all plant movement counts as behaviour (or decision making for that matter) any more than all animal movement does. Knee-jerk reactions are excluded, for example. Rather, we are claiming that there are more ways to move than locomotion.

To see this more clearly, consider the well-studied example of *Physarum polycephalum* (aka ‘slime mould’). *P*. *polycephalum* is a unicellular protist which has received much attention for the complex behaviour it shows during its multinucleate plasmodial phase. At this stage, slime mould consists of a network of tubules which carry protoplasm throughout the entire organism courtesy of a series of oscillators that pulse, expanding and contracting the tubules, depending on external circumstances and the state of the nearby oscillators. When the organism detects an attractant, pulses nearest to the attractant increase, causing the organism to grow towards it. The opposite occurs when the organism detects a repellent: activity of the oscillators decreases, reducing the flow of protoplasm in this area.

Not unlike plants, *P. polycephalum* has been tested in multiple protocols adapted from human and animal decision-making studies [6]. These experiments have shown that slime mould compares the relative properties of multiple options in making choices [54] in that it can discriminate high-calorie over low-calorie food, and that it can make sophisticated trade-offs when access to some nutrient source involves exposure to danger [55,56]. More strikingly, it has been reported that slime mould is susceptible to some biases previously observed in human and non-human animals [57]. Overall, these studies reinforce the view that brainless organisms can sample and integrate information from different internal and external parameters in order to make adaptive decisions. As Smith-Ferguson and Beekman explicate, ‘[t]he coupling of neighbouring oscillators means information can be encoded or “entrained” into oscillation frequencies and transferred to parts of the plasmodium which are too far to detect the chemical cues. Hence, the physiology of the organism—its fluid dynamics—allows it to transfer information throughout the organism without the need for a nervous system’ [58] (p. 467). Locomotion is not here considered a necessary condition for behaviour and decision making.

In summary, the relevant (functional) analogy holds between bacteria, plants, and other organisms such as protists. If we grant idiosyncratic forms of behaviour selection in different organisms, it becomes easier to accept decision making in plants. In other words, if we (i) accept minimal decision-making abilities in taxa such as prokaryotes and protists alongside (ii) movement via growth, the argument for extending decision-making abilities to plants is strengthened. Alternatively, pressure is placed on the sceptics of plant decision making to either deny decision making in bacteria (and protists) or demonstrate some non-arbitrary difference between the former and plant behaviour.

## 4. Future Research

Research on bacteria suggests that prokaryotes may serve as ‘experimental organisms’ for studying decision making more broadly (up to the level of non-conscious human decision making), with an emphasis placed on the fact that discovering the ability in question in simpler organisms assists in revealing the core characteristics of the mechanisms underlying that phenomenon. For example, Huang et al. [19] argue that by identifying mechanisms for decision making in these (relatively) simple cases, we may gain insight into the mechanisms for decision making in more prototypical cases, as in humans and other animals (p. 1064). As we have seen, this lesson extends beyond prokaryotes to include other ‘minimal’ decision makers (see the example of slime moulds, which are eukaryotic), with the potential to include plants. It goes without saying that the specific mechanisms will vary by necessity. In the aforementioned illustration of root growth behaviour, different volatiles may serve to modulate cellular membrane properties at the root apex, which in turn would explain the differential distribution of the plant hormone auxin that results in the positive or negative tropism exhibited [35]. Yet at a higher level of description, membrane properties will serve to identify common threads, as plant–animal comparative electrophysiology reveals [18]. The response to anaesthesia by both animals and plants, whereby the integrity of the plasma membrane is compromised with the alteration of key membrane properties [59,60] provides a clear-cut illustration of this.

A comparison of traits across different taxa may also offer insight into the evolutionary history of decision making. As Petrillo and Rosati [61] write ‘the broad lesson is that evolutionary explanations for a given species’ pattern of decision-making need to account for how that strategy plays out for specific species in their specific ecological context’ (p. 780). Using the example of diverging preferences in decision making about the temporal and spatial distribution of rewards in cotton-top tamarins and common marmosets, the authors go on to note that ‘[e]mpirical evidence from comparative studies suggests that some differences in species decision-making strategies map onto differences in these species’ wild ecology’ (p. 781). Whilst De Petrillo and Rosati are concerned with comparative animal cognition, we can see how their comparative method might apply, on a greater scale, across the tree of life.

Promising insights from studying decision making in experimental organisms, such as bacteria and plants, for our understanding of decision making in more prototypical cases, such as humans and other animals, itself provides justification for attributing genuine decision-making abilities to the experimental organisms. If studying abilities in experimental organisms that resemble decision making in prototypical cases, such that research in the former leads to discoveries in the latter, then we should consider recognising that the experimental organisms possess that ability. Or more pragmatically, by treating organisms such as bacteria and plants as capable of making choices, we gain insight into less contested cases of decision making in other organisms. Ultimately, one may fear that any refusal to rubber-stamp the decision-making credentials of bacteria or plants reflects a mere semantic (but potentially unhelpful) preference if bacteria and plant processes do resemble paradigmatic decision making to the extent that the former guides discoveries about the latter (for related discussion see [62]).

The search for decision making in plants may further expand our use of non-neural taxa for the identification of key components in decision making across the tree of life. In addition to engaging with the broader philosophical debate around the extension of psychological predicates, future work should further detail the control mechanisms for plant decision making and the potential of plants as experimental organisms, whilst also still exploring how plants make choices by idiosyncratic, plant-specific means.

## 5. Conclusions

We should take seriously the possibility that plants make choices. This paper presented recent research that evidences decision making in bacteria, thus supporting the broader notion that decision making does not require a centralised system for processing information. However, one might think there is a breakdown in the analogy between plants and bacteria because only the latter select between an array of genuine behaviours; in particular, plants do not move. We argued that we ought to accept that plants behave in the same sense as bacteria (*mutatis mutandis*) because plants do move, albeit at a slower timescale than most animal movements and primarily via growth. If we accept decision making in bacteria, and we accept that plants select between movements in response to their environment, then we have firm grounds to accept that plants make decisions.

## Data Availability

The data is contained within the manuscript.
